# Conventional epidemiology underestimates the incidence of asthma and wheeze-a longitudinal population-based study among teenagers

**DOI:** 10.1186/2045-7022-2-1

**Published:** 2012-01-04

**Authors:** Linnéa Hedman, Anders Bjerg, Bo Lundbäck, Eva Rönmark

**Affiliations:** 1 The OLIN-studies, Norrbotten County Council, S-971 89 Luleå, Sweden; 2 Department of Public Health and Clinical Medicine, Occupational and Environmental Medicine, Umeå University, S-901 87 Umeå, Sweden; 3 Krefting Research Centre, Institute of Medicine, The Sahlgrenska Academy, University of Gothenburg, S-405 30 Gothenburg, Sweden

**Keywords:** asthma, adolescents, epidemiology, incidence, study design, wheezing

## Abstract

**Background:**

Because of shifts in the gender ratio and incidence and remission rates of asthma during the teen ages, the methodology of incidence studies among teenagers is important, i.e. if the time intervals between surveys are too long, the incident cases might not be properly identified. The aim was to study the impact of study design on the incidence rates of asthma and wheeze during the teen ages.

**Methods:**

In a study about asthma and allergic diseases within the OLIN studies (Obstructive Lung Disease in northern Sweden), a cohort of school children (n = 3,430) was followed annually by questionnaire from age 8 yrs. In the endpoint survey (age 18 yrs) 2,582 (75% of original responders) participated. Incident cases from age 12-18 yrs were identified by two methods: annual questionnaire reports (AR) and baseline-endpoint surveys only (BE).

**Results:**

The cumulative incidence of asthma and wheeze was significantly higher based on AR compared to BE. Compared to the incidence rates based on all the annual surveys, the calculated average annual rates based on BE were in general lower both among the boys and among the girls. There were no differences between boys and girls in incidence rates of asthma or wheeze during the early teen years. However, from the age of 15 years, the annual incidence rates were significantly or borderline significantly higher among girls than boys. At onset, the additional cases of current asthma identified by AR had significantly less severe asthma than those identified in BE (p < 0.02).

**Conclusion:**

the size of the incidence of asthma and wheeze during the teen ages was influenced by study design. By using the conventional prospective study design with longer follow-up time, the incidence was underestimated.

## Background

The number of population-based studies about the incidence of asthma and wheeze during adolescence are still limited [1-6]. In the majority of these studies, an average annual incidence rate was estimated based on two surveys, several years apart. Hence, the present knowledge of the annual incidence rate of asthma and wheeze is based on extrapolations from the cumulative incidence, and so far, little is known about the true annual incidence rate during adolescence.

Despite considerable differences in the methodology and definitions, the available studies show that the incidence of asthma is highest in early childhood and decreases in adolescence. In prospective studies, the incidence of asthma has been reported at 0.6-1.1/100 per year in pre-teenagers [7] and teenagers [1,2,8]. Between 16 to 22 years of age, a prospective study from Finland reported a low incidence of 0.2/100 per year [9], while an American study reported 1.3/100 per year years [5]. Compared to most of these prospective studies, lower incidence rates have been reported both in retrospective [10,11] and register studies [12].

Before adolescence, the prevalence of asthma and wheezing is usually higher among boys than girls, while in adults this association is reversed [1,10,13,14]. This shift occurs during early teenage due to higher incidence of asthma among girls than boys [15], and possibly lower remission among girls than boys [16]. During adolescence, some individuals with asthma experience improvement in severity of symptoms or even remission, but relapse in early adulthood is common [14,17]. Because of the complex natural history of asthma, these changes during adolescence may impact differently on the estimated incidence in prospective studies, depending on the methodology. However, no study has reported the consequences of the frequency and time intervals between surveys on the incidence rates of asthma and wheeze among teenagers. The aim of the present study was to study the influence of study design on the incidence rates of asthma and wheeze during the teen ages.

## Methods

### Study design and subjects

The OLIN paediatric study I, is a longitudinal study about asthma, rhinitis, eczema, and allergic sensitization among schoolchildren in Northern Sweden. The overall aims and methods have previously been described in detail [13]. The parents of all 3,525 children aged 7 and 8 years in three municipalities, enrolled in the first and second grades in 1996, were invited to complete a questionnaire. The participants (n = 3,430; 97%) formed a cohort that was followed by annual questionnaires until high school graduation in 2006 and 2007, respectively. Participation in the questionnaire surveys between 1996 and 2006 are presented in an online supplement. The study population in this paper consisted of the 75% of the cohort (n = 2,582 children, 50% boys) who participated in 1996 and again in 2006 at the age of 18 years. The prevalence of asthma and wheeze at recruitment in 1996 was similar among the study population and those who had dropped out. We have in previous publications reported on the incidence of asthma and wheeze between the ages 8 (1996) up to 12 years (2000) [7,18]. In the present paper, the incidence of asthma and wheeze during the teen years was studied, from 12 to 18 years of age. The study was approved by the Ethics Committee at Umeå University, Sweden. Informed consent was given by parent or guardian at the beginning of the study.

### Questionnaire

The questionnaire included the International Study of Asthma and Allergies in Childhood (ISAAC) core questionnaire [19], additional questions about physician diagnoses of asthma and allergic diseases and symptoms, and possible risk factors [20]. Until age 12 years, the questionnaire was completed by the parents. From 13 years of age and onward, the teenagers completed the questionnaire at school. Evaluations about this methodological change have been performed by comparing parentally and self-completed questionnaires from the survey in 2002 (age 13-14 years). The results showed good agreement between parentally and self-completed questionnaires in questions about allergic diseases and environmental factors [21], and in questions about asthma and wheeze [22].

### Statistical analyses and data management

Analyses were made using the computer software PASW Statistics (Version 18.0; SPSS Inc, Chicago IL, USA). For assessment of sex differences in the incidence of asthma, χ^2^-tests were used and a p-value < 0.05 was considered statistically significant. Main outcome was the incidence of *ever asthma, physician-diagnosed asthma, current asthma *and *current wheeze *(for definitions, see Appendix 1). An arbitrary score of asthma severity ranging from 0-5 was developed as described by Andersson et al [23]. It included *current wheeze*, daily use of asthma medication, ≥ 1 night per week with disturbed sleep, at least one episode of speech-limiting wheeze, and > 12 episodes of wheezing. Each item should have occurred during the last 12 months and yielded one point each. Calculations of the incidence were performed by four methods;

1. annual incidence rate based on all the annual reports (AR). Incident cases identified in each annual survey were excluded from the population at risk in the subsequent surveys.

2. estimated annual incidence rate based on the baseline and endpoint reports only (BE).

3. cumulative incidence based on AR, i.e. those reporting asthma/wheeze in any of the questionnaire surveys between 2001 and 2006.

4. cumulative incidence based on BE, i.e. those reporting asthma/wheeze in the 2006 survey.

**Figure 1:**
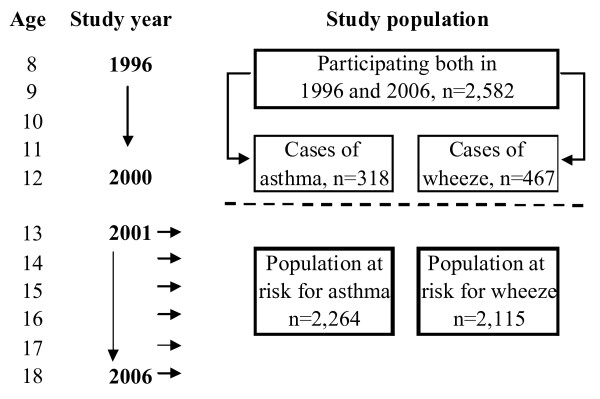
**Flow chart of the identification of the population at risk for asthma and wheeze based on annual questionnaire surveys**.

The incidence rate was calculated as: $\frac{a}{Years\times \left(b-\left(a∕2\right)\right)}$ where *a *is the incident cases and *b *the population at risk at baseline. The cumulative incidence was calculated as: *Incident cases/Population at risk*. The population at risk for asthma excluded those who had reported physician-diagnosed asthma or ever asthma in any of the questionnaire surveys in 1996-2000 (age 8 to 12 years) [7], or in a validation study performed in 1997 were identified as having asthma [24]. The population at risk for wheeze excluded those who had reported current wheeze in any of the questionnaire surveys in 1996-2000. Of the study population (n = 2,582), 12.3% (n = 318) had reported asthma and 18.1% (n = 467) current wheeze at any time before the age of 13 years (Figure 1). Thus, at baseline the population at risk for asthma consisted of 2,264 teenagers (51% girls), and the population at risk for wheeze was 2,115 teenagers (51% girls). Risk factor analyses for the incidence of current asthma and current wheeze based on AR and BE respectively, were performed by multiple logistic regression analyses adjusting for sex, family history of asthma and physician-diagnosed rhinitis. The results were expressed as odds ratios (OR) with 95% confidence intervals (CI).

## Results

### Annual incidence rate

**Figure 2:**
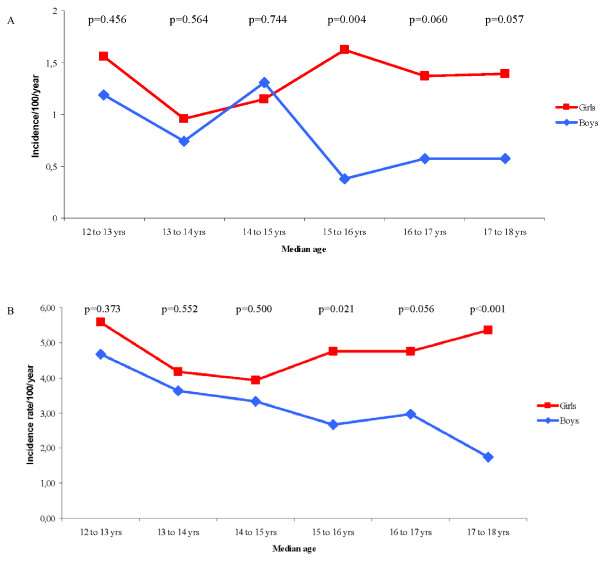
**A: Annual incidence rates of physician-diagnosed asthma from age 12 to18 years by sex, based on annual reports**. B: Annual incidence rates of current wheeze from age 12 to 18 years by sex, based on annual reports.

Based on AR, there were no differences between boys and girls in incidence rates of *physician-diagnosed asthma *or *current wheeze *during the early teen years (Figure 2a and 2b). However, from the age of 15 years, the annual incidence rates were significantly or borderline significantly higher among girls than boys. Corresponding analyses for *ever asthma *and *current asthma *showed similar results. The annual rates varied between 0.7-2.6 and 0.6-1.5/100 per year, respectively, among the boys and between 1.2-3.1 and 1.1-1.8/100 per year, respectively, among the girls (Additional file 2).

Based on the incident cases identified by BE, the average annual incidence was calculated (Table 1). Compared to the incidence rates of *current wheeze *based on all the annual surveys (Figure 2b), the calculated average annual rates were lower both among the boys and among the girls. A similar pattern was found for the asthma variables (Figure 2a; Additional file 2).

**Table 1:** Average annual incidence rate (cases/100/year) of asthma and wheeze from the age of 12 to 18 years based on the baseline and endpoint (BE) reports only.

	Boys		Girls	
	
	(n)	Incidence rate	(n)	Incidence rate
Ever asthma	(66)	1.03	(109)	1.64
Physician-diagnosed asthma	(50)	0.78	(79)	1.17
Current asthma	(38)	0.59	(67)	0.99
Current wheeze	(61)	1.02	(120)	1.95

### Cumulative incidence

**Figure 3:**
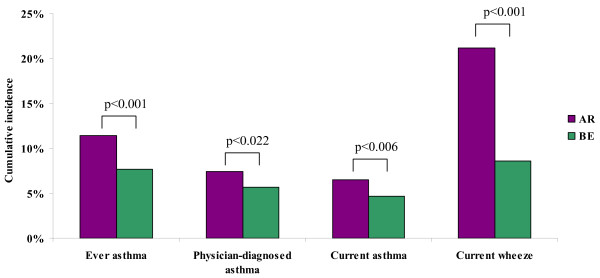
**Cumulative incidence (%) of asthma and wheeze from age 12 to 18 years based on annual reports (AR) and the baseline and endpoint surveys only (BE), respectively**.

The cumulative incidence of asthma was consistently and significantly higher when based on AR, compared to identification of incident cases by BE only (Figure 3). In both AR and BE methodology, the cumulative incidence of asthma and wheeze was significantly higher among girls than boys (Table 2).

**Table 2:** Cumulative incidence (%) of asthma and wheeze between ages 12 to 18 years by method of measure.

		Boys		Girls		
			
Condition	Method	(n)	Incidence %	(n)	Incidence %	Difference p-value
Ever asthma	AR	(100)	9.09	(158)	13.57	< 0.001
	BE	(66)	6.00	(109)	9.36	0.003
Physician-diagnosed	AR	(64)	5.82	(103)	8.85	0.006
asthma	BE	(50)	4.54	(79)	6.79	0.021
Current asthma	AR	(56)	5.09	(91)	7.82	0.008
	BE	(38)	3.45	(67)	5.76	0.009
Current wheeze	AR	(178)	17.33	(270)	24.82	< 0.001
	BE	(61)	5.94	(120)	11.03	< 0.001

AR: Incidence based on the annual reports

BE: Incidence based on the baseline and endpoint surveys only

### Asthma severity score

**Figure 4:**
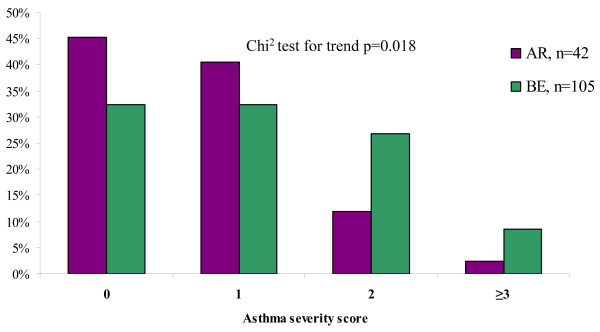
**The distribution of asthma severity among incident cases of current asthma identified in the annual reports (AR) and based on the baseline and endpoint surveys only (BE), respectively**.

The BE methodology identified 105 incident cases of *current asthma *(Table 2). Additionally, another 42 incident cases were identified by the AR method, yielding totally 147 cases. An asthma severity score based on the first of the surveys they reported *current asthma *was calculated in order to study whether the additional 42 AR cases differed in asthma severity from the cases identified by BE. The incident cases identified by BE had significantly higher scores than the AR cases (p < 0.02) (Figure 4). The prevalence of allergic sensitization and physician-diagnosed rhinitis was similar in the two groups. Among the incident cases of *current asthma *there was no statistically significant difference in asthma severity by sex.

### Multivariate analyses

Based on the incident cases identified by AR and BE, respectively, risk factor analyses were performed by multiple logistic regression analyses (Table 3). Female sex, family history of asthma and physician-diagnosed rhinitis were all significantly related to increased incidence of *current asthma *and *current wheeze *with only one exception: the association between family history of asthma and *current wheeze *which was borderline significant. Compared to AR, the analyses based on the incident cases identified by BE yielded similar but slightly higher odds ratios and wider confidence intervals.

**Table 3:** Risk factor analyses for the incidence of current asthma and current wheeze from age 12-18 years, by method of measure, expressed as odds ratios (OR) with 95% confidence intervals by multiple logistic regression analyses.

	*Dependent variables*							
	
	Current asthma				Current wheeze			
	
	AR		BE		AR		BE	
	
*Independent variables*	OR	95% CI	OR	95% CI	OR	95% CI	OR	95% CI
Female sex	1.65	1.17-2.34	1.81	1.20-2.73	1.52	1.24-1.88	2.06	1.49-2.85
Family history of asthma	1.54	1.05-2.25	1.76	1.14-2.71	1.26	0.98-1.62	1.44	1.01-2.06
Physician-diagnosed rhinitis	3.13	1.97-4.98	2.91	1.69-5.02	1.97	1.37-2.84	2.59	1.62-4.14

AR: Incidence based on the annual reports

BE: Incidence based on the baseline and endpoint surveys only

## Discussion

This study has clearly shown the impact of study design on the measure of the incidence of asthma and wheeze during adolescence. The incidence may be underestimated if the follow-up time between surveys is too long. In this population-based prospective study, the incidence of asthma and wheeze estimated from baseline to endpoint was significantly lower than that obtained by the annual reports. While the incidence rates were similar between boys and girls during the early teen years, from the age of 15 years the annual rates in general became significantly higher among the girls. The additional cases of current asthma identified in the annual reports generally had milder symptoms compared to the cases identified at endpoint only. Thus, the conventional baseline-endpoint methodology underestimated the true annual incidence.

The study design of the OLIN paediatric study I allowed for a comparison of incidence estimates obtained by using different times to follow-up. The use of annual follow-ups increases the precision in the incidence estimates. However, to date, this is the first study that has measured both the true and the average annual incidence rate of asthma and wheeze during the teen years in the same study population. Conventionally, the average annual incidence is calculated based only on baseline and endpoint surveys with varying follow-up times. Depending on the definition of asthma, the average annual incidence rate of asthma based on the BE methodology in the present study was similar to other studies among teenagers that have reported rates of 0.6-1.3/100 per year [1,2,5,8]. However, compared to the true annual incidence rate obtained by the AR methodology, the average annual incidence rate of asthma and wheeze was underestimated. For instance regarding current wheeze, the average annual incidence rate based on BE among the boys was 1.0/100 per year and among the girls 1.9/100 per year, while the real annual incidence rate based on AR varied from 1.8 to 4.7/100 per year among the boys and from 3.9 to 5.6/100 per year among the girls.

It has been suggested that in prospective studies, recurrent surveys may increase the reporting of symptoms and cause an overestimation of the prevalence and incidence. However, when the prevalence of asthma and respiratory symptoms from a longitudinal study was compared to those obtained from a cross-sectional study within the same population in New Zealand, no difference was found [25]. In the present study, the incidence was consistently higher when the incident cases were identified by the recurrent surveys compared to the study design with longer follow-up time. For instance, the AR methodology identified 40% more incident cases of current asthma than the BE approach. Thus, there were incident cases identified in the annual surveys that did not report having asthma or wheeze, respectively, in the endpoint survey. These additional cases of incident asthma had significantly lower asthma severity scores compared to the BE cases. Nevertheless, they were classified as having current asthma based on strict criteria, including physician-diagnosis and in addition having either wheeze or using asthma medication during the last 12 months. Furthermore, the prevalence of allergic sensitization and physician-diagnosed rhinitis did not differ between these two groups of incident cases. Thus, nearly one-third of the actual incident cases of current asthma were not properly identified due to having a mild asthma and short time between onset and remission. On the other hand, the risk factor analyses based on the AR method yielded somewhat lower odds ratios for well known risk factors for asthma compared to corresponding analyses based on BE, indicating that the AR method includes less specific cases. An over-diagnosis of asthma among teenagers may have contributed to these results.

Compared to prospective studies, retrospective studies more often show lower incidence rates. In a British cohort followed from birth to age 33 years, the incidence of asthma or wheeze was 1.3/100 per year when studied prospectively, while retrospectively in the same cohort it was 0.9/100 per year [10]. In our cohort, the prevalence of asthma and wheeze according to the survey at age 11-12 year was lower compared to when the prevalence was based on all annual reports from age 7-8 to 11-12 years [26]. Further, it has been shown that the incidence of asthma decreased by time from diagnosis [11]. Similar to the retrospective design, prospective studies with longer follow-up time would also be affected by recall bias, as supported by our findings.

The largest difference in incidence between study designs was found for current wheeze, where the AR methodology identified 48% more cases than the BE methodology. This result was expected, as wheeze is more transient than established asthma, and current wheeze was defined as any wheeze during the last 12 months. There are many other causes of wheeze than asthma among teenagers, for instance respiratory infections and poor physical condition [27].

Regardless of study design, the cumulative incidence of asthma and wheeze up to age 18 years was significantly higher among girls than boys, in accordance with other studies [1,2,8]. In young children, both the prevalence and incidence of asthma and wheeze are usually higher among boys than girls, while in adults the associations are reversed [1,10,13,14]. Although it has not been established exactly when, this shift occurs during the teen years, in part due to higher incidence of asthma among girls than boys [15]. One study suggests that the prevalence shift starts at the age of 11 and goes on until age 16 years [16]. In the present study we demonstrated that the incidence rates were still similar during the early teen years. However, as the annual rates became significantly or borderline significantly higher among girls from the age of 15 years, we suggest that this is the age when the shift occurs.

The strengths of this study are the prospective design and the high participation rates with the possibility to follow more than 75% of the original cohort from the age of 7 to 18 years. Further, the questionnaire included the validated and internationally used ISAAC protocol [19]. The design of the questionnaires and the method of distribution were identical every year. Initially in this cohort, the questionnaires were completed by the parents, but from the age of 12 years, the teenagers were the responders. Thus, identification of incident cases in the present study was based only on self-reporting of asthma and wheeze. A limitation is the lack of objective data to validate the asthma diagnosis. However, the validity of the questionnaire responses was verified by a comparison of parentally and self-completed questionnaires at the age of 13-14 years which showed very good agreement for questions about asthma [21,22]. A previous validation study of the question about physician-diagnosis of asthma performed at the age of 8-9 years, showed very high specificity [24]. Since parental and teenager reports of asthma were similar at age 13-14 years [22], it can be assumed that the specificity is still high.

In conclusion, the size of the incidence of asthma and wheeze during the teen years was influenced by study design. By using the conventional prospective study design with longer follow-up time, the incidence was underestimated. Study design and follow-up time is important to consider when the incidence of asthma and wheeze is compared between studies.

## List of abbreviations

AR: Incidence based on all the annual reports; BE: Incidence based on the baseline and endpoint surveys only; CI: Confidence Interval; ISAAC: International Study of Asthma and Allergies in Childhood; OLIN: The Obstructive Lung Disease in Northern Sweden studies; OR: Odds Ratio.

## Competing interests

The authors declare that they have no competing interests.

## Authors' contributions

LH contributed to the design of the study, participated in data collection, performed statistical analyses, and drafted the manuscript. AB participated in data collection, participated in analysing and interpreting of data, and helped draft the manuscript. BL contributed to the design of the study, participated in analysing and interpreting of data, and helped draft the manuscript. ER conceived of the study, participated in data collection, participated in analysing and interpreting of data, and helped draft the manuscript. All authors read and approved the final manuscript.

## Appendix 1

The definitions of asthma and wheeze were based on the following questions:

*Ever asthma: *Have you ever had asthma?

*Physician-diagnosed asthma: *Have you been diagnosed by a physician as having asthma?

*Current asthma: *Physician-diagnosed asthma, and either current wheeze or use of asthma medication during the last 12 months.

*Current wheeze: *Have you had wheezing or whistling in the chest in the last 12 months?

## Supplementary Material

Additional file 1 **Table including data on the participation in the OLIN pediatric study I in 1996, 2000 and from 2001 to 2006**. Click here for file

Additional file 2 **Table including data on annual incidence rate of asthma from age 12 to 18 years by sex, based on the annual reports**. Click here for file
