# Supplementary material for: Conventional epidemiology underestimates the incidence of asthma and wheeze-a longitudinal population-based study among teenagers
Source: Clin Transl Allergy. 2012 Jan 4;2:1. doi: 10.1186/2045-7022-2-1 (PMC3395824; doi:10.1186/2045-7022-2-1)
Supplement: Additional file 1 — Table including data on the participation in the OLIN pediatric study I in 1996, 2000 and from 2001 to 2006. [file 2045-7022-2-1-S1.PDF]

Additional file 1. Participation in the OLIN paediatric study in 1996, 2000 and from 2001 to 2006.

Year	Mean age	Invited	Participated	%
1996	8	3,525	3,430	97%
2000	12	3,229	3,151	98%
2001	13	3,192	2,941	92%
2002	14	3,142	3,013	96%
2003	15	3,134	2,989	95%
2004	16	3,097	2,985	96%
2005	17	3,012	2,805	93%
2006	18	2,796	2,582	92%
