# Supplementary material for: Conventional epidemiology underestimates the incidence of asthma and wheeze-a longitudinal population-based study among teenagers
Source: Clin Transl Allergy. 2012 Jan 4;2:1. doi: 10.1186/2045-7022-2-1 (PMC3395824; doi:10.1186/2045-7022-2-1)
Supplement: Additional file 2 — Table including data on annual incidence rate of asthma from age 12 to 18 years by sex, based on the annual reports. [file 2045-7022-2-1-S2.PDF]

Additional file 2. Annual incidence rate (cases/100/year) of ever asthma and current asthma from the age of 12 to 18 years by sex, based on annual reports.

		Boys		Girls		
Condition	Median age	(n)	Incidence	(n)	Incidence	Difference by sex p-value
Ever asthma	12 to 13 yrs	(28)	2.58	(36)	3.14	0.432
	13 to 14 yrs	(14)	1.31	(27)	2.42	0.059
	14 to 15 yrs	(15)	1.43	(13)	1.19	0.626
	15 to 16 yrs	(7)	0.67	(21)	1.95	0.011
	16 to 17 yrs	(8)	0.78	(23)	2.18	0.008
	17 to 18 yrs	(10)	0.98	(21)	2.03	0.051
Current asthma	12 to 13 yrs	(10)	0.91	(14)	1.21	0.495
	13 to 14 yrs	(11)	1.01	(13)	1.14	0.780
	14 to 15 yrs	(16)	1.49	(13)	1.15	0.482
	15 to 16 yrs	(6)	0.57	(20)	1.80	0.009
	16 to 17 yrs	(4)	0.38	(14)	1.28	0.023
	17 to 18 yrs	(9)	0.86	(17)	1.57	0.136
